# Effects of Additional Mesopores and the Surface Modification of the Y-Type Zeolite on the Alkane Oxidation Activity of Iron Complex-Encapsulated Catalysts

**DOI:** 10.3390/molecules30040966

**Published:** 2025-02-19

**Authors:** Takamasa Takeda, Masaya Okamura, Syuhei Yamaguchi, Hidenori Yahiro, Shiro Hikichi

**Affiliations:** 1 Department of Applied Chemistry, Faculty of Chemistry and Biochemistry, Kanagawa University, 3-27-1 Rokkakubashi, Kanagawa-ku, Yokohama 221-8686, Japan; r202270140iq@jindai.jp; 2 Department of Materials Science and Biotechnology, Graduate School of Science and Engineering, Ehime University, 3 Bunkyo-cho, Matsuyama 790-8577, Japan; yamaguchi.shuhei.mz@ehime-u.ac.jp (S.Y.); hyahiro@ehime-u.ac.jp (H.Y.)

**Keywords:** zeolites, mesoporous materials, hydrophobic effect, immobilized catalyst, oxidation

## Abstract

Catalytic alkane hydroxylation activities of the iron complex encapsulated into the micropore of the Y-type zeolite and mesoporous zeolites, the latter of which were obtained by the partial removal of aluminum and alkaline treatment, have been explored by using H_2_O_2_ as the oxidant. The iron complex with tris(pyridylmethyl)amine (=TPA) encapsulated into the micropore of the genuine Y-type zeolite was a more stable and effective cyclohexane hydroxylating heterogeneous catalyst compared to the corresponding copper analogue as well as the non-encapsulated homogeneous Fe-TPA complex. The chemical modification of the zeolite supports with the organic groups led to changing the catalytic activity depending on the size and the hydrophobic or hydrophilic nature of the added organic groups. When the content of water in the solvent was increased, the activity of the hydrophilic longer chain-modified catalyst was improved compared to that applied on the reaction with the non-aqueous solvent. The hydrophobic fluoroalkyl modifier located near the entrance of the micropore hindered the access of the substrate and aqueous H_2_O_2_ to the encapsulated iron complex site in the genuine Y-type zeolite. On the other hand, the hydrophobic modification effectively improved the activity of the catalyst with the zeolite support having higher amounts of mesopores. The synergistic effect of the wider bore diameters and the hydrophobic nature derived from the fluoroalkyl chains led to the concentration of the hydrocarbon substrate near the active iron complex.

## 1. Introduction

In homogeneous liquid phase catalytic reactions, the deactivation of metal complex catalysts often occurs due to intermolecular reactions caused by the generated reactive species. The encapsulation of metal complex molecular catalysts into micropores of zeolites is an effective way to avoid the intermolecular reactions leading to such deactivation [1]. For example, the copper(II) hydroperoxide species generated by the reaction of a mononuclear copper complex isolated in the 3-D micro-space of a zeolite with hydrogen peroxide is significantly more stabilized than in the solution state, and this species works as an active oxidant for organic substrates [2]. The encapsulation of metal complex catalyst molecules into the micropores of zeolites is also expected to reveal the intrinsic reactivity of the complex molecules, which is interesting as a fundamental study of coordination chemistry and catalytic chemistry.

**Figure 1:**
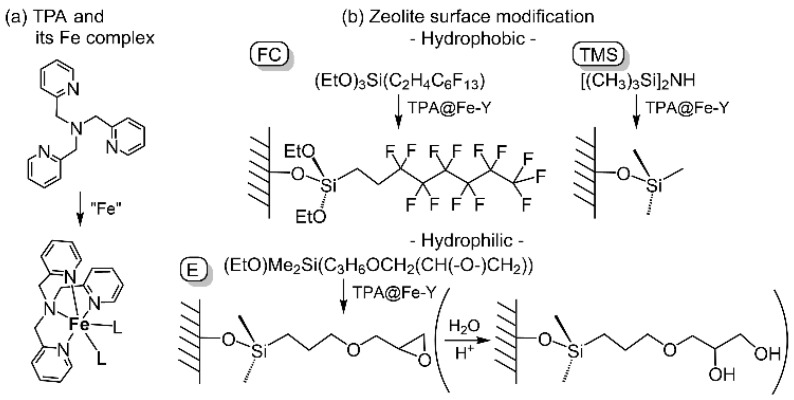
Schematic drawing for TPA and its iron complex (**a**) and zeolite surface modification by hydrophobic and hydrophilic groups (**b**) demonstrated in this work.

In this context, we have investigated the catalytic activity of a ship-in-a-bottle catalyst consisting of an iron complex with tris(pyridylmethyl)amine (TPA; Figure 1a) ligand encapsulated in a Y-type zeolite super cage, which has been studied as a model for non-heme iron enzymes catalyzing the oxygenation of hydrocarbons. TPA and its derivatives have been employed to model compounds of non-heme metal oxygenases [3,4,5,6,7,8]. Extensive investigations on the chemistry of copper–oxygen complexes revealed that some dinuclear and mononuclear copper species exist in equilibrium [7]. In addition, both the dinuclear and mononuclear iron-active oxygen complexes with TPA derivative ligands have been reported [3,4,5,6,7,8,9,10,11,12,13,14]. When M-TPA species would be encapsulated in the micropores of zeolites, only the mononuclear complexes could exist due to the limitation of space. Therefore, the intrinsic properties and reactivity of the mononuclear species would be able to be revealed. To date, several examinations of the cyclohexane oxygenation with H_2_O_2_ by the ship-in-a-bottle catalysts encapsulating the iron complexes other than the TPA ligand one have been reported [15,16,17,18,19]. In this study, we have examined the catalytic performance of the prepared ship-in-a-bottle catalysts based on M-TPA complexes (M = Fe, Cu).

When immobilized metal complex catalysts are used as heterogeneous catalysts for the transformation of organic molecules in the liquid phase, the accessibility of the substrate to the catalytic active site is reduced. Especially in the case of the ship-in-a-bottle-type catalysts, where the catalytic active site is constructed inside the micropores, diffusion of the substrate into the interior of the zeolite is difficult, and only the micropores near the outer surface of the zeolite particles can serve as an effective reaction site. A possible way to solve this problem is by adding mesopores by treating the zeolite particles in an appropriate manner. This allows the substrate to easily approach not only the active sites close to the surface of the zeolite particles but also the sites facing the mesopore [20,21,22,23,24,25,26,27,28]. In this study, therefore, mesoporous zeolites were also applied as the support of the ship-in-a-bottle-type catalysts.

The chemical modification of the surface of the inorganic support with organic materials is another effective strategy to improve catalytic performance by controlling the affinity of solid catalyst particles with solvents and substrates. As we have demonstrated, the hydrocarbon oxygenation activities of the immobilized iron complex catalysts are enhanced by the modification of the support wall with long-chain alkyl fluoride [29,30]. Also, we have found that the hydrophobic or hydrophilic nature of the support leads to drastic changes in the catalytic reaction selectivity. The tungstate species immobilized on the organic cation-modified mesoporous silica support catalyzes alkene epoxidation with H_2_O_2_ in the less-polar solvents, and the activity of the hydrophilic catalyst which retains silanol groups is higher than the hydrophobic one. On the contrary, the hydrophobic catalyst mediates not only the epoxidation but also the hydration to give the corresponding diol in H_2_O [31]. Building upon these successes, we have explored the effect of the chemical modification on the surface silanol groups by using hydrophobic or hydrophilic compounds (Figure 1) for the Y-type zeolite support of the ship-in-a-bottle-type catalysts as well.

## 2. Results and Discussion

### 2.1. Preparation, Characterization and Activity of the Catalysts with Unmodified Zeolite Y

An iron ion-exchanged Y-type zeolite, Fe-Y, was prepared by replacing some of the Na ions in Na-ion-containing Y-type zeolite Na-Y (purchased from FUJIFILM WakoChemical Corp., Kanagawa, Japan) with FeSO_4_. The chelating ligand TPA reacted with Fe-Y to yield the corresponding ship-in-a-bottle-type catalyst TPA@Fe-Y. In this study, the copper analogue TPA@Cu-Y was also prepared by the same procedures. The copper ion-exchanged zeolite, Cu-Y, was prepared from Cu(NO_3_)_2_∙3H_2_O and Na-Y [2]. The resulting Cu-Y was treated with TPA. The loading amounts of the metal ion were determined by atomic absorption spectrometry and the amounts of TPA in the catalysts were estimated by thermogravimetric analysis. Fort both the prepared iron and copper catalysts TPA@M-Y (M = Fe, Cu), the loading ratios of M:TPA were almost 1:1 (Table 1). When mesoporous zeolites Y_m1 and m2 were used as the supports, the loading ratio of iron to TPA exhibited similar trends (see Section 2.2). UV-vis spectral analysis supported the formation of the corresponding metal complexes with TPA (Figure S1). In TPA@Cu-Y, a d-d transition band of copper(II) appeared around 700 nm. In the iron catalyst, broad absorption bands were observed in a 350–450 nm region in addition to the strong bands in the UV region attributed to the aromatic rings of TPA.

**Figure 2:**
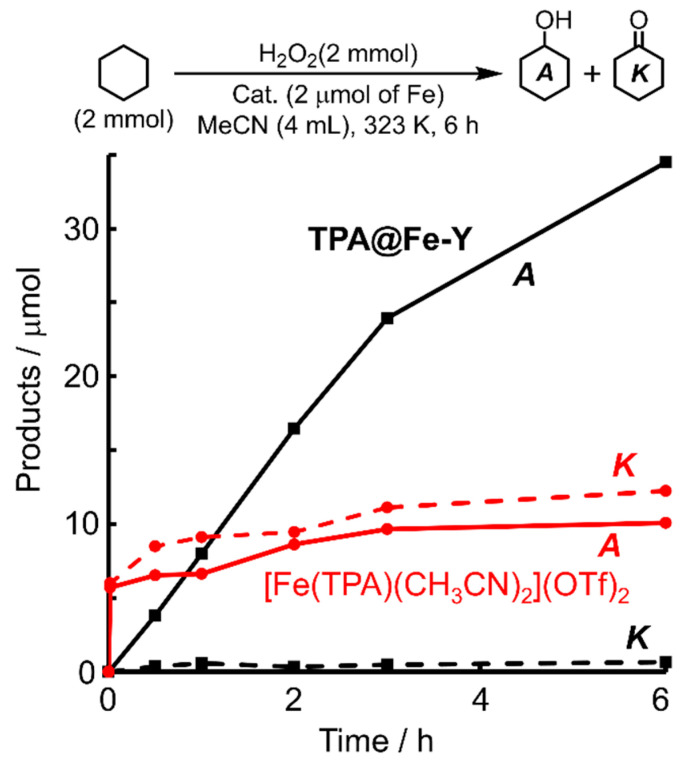
Catalytic performances of the heterogeneous catalyst TPA@Fe-Y and the homogeneous catalyst [Fe(TPA)(CH_3_CN)_2_](OTf).

The catalytic activity of the prepared TPA@M-Y (M = Fe, Cu) toward cyclohexane oxidation with H_2_O_2_ was explored. The iron catalyst produced alcohol immediately, and its selectivity was kept high even at 24 h (Figure S2). From the resulting reaction solution, only a negligible amount of iron was detected by atomic absorption analysis. The activity of TPA@Fe-Y was clearly different from that of the homogeneous prototype [Fe(TPA)(CH_3_CN)_2_](OTf)_2_ [5,29]. In the homogeneous system, the immediate formation of alcohol and ketone occurred concomitant with the generation of O_2_ bubbles due to decomposition of H_2_O_2_. On the other hand, the heterogenaized iron catalyst yielded alcohol with high selectivity. Both TON and A/K values were higher for TPA@Fe-Y at 2 h as shown in Figure 2. Therefore, the encapsulation of the Fe-TPA complex in the micropore enhanced the stability of the complex and made it possible to progress the oxidation reaction continuously.

**Figure 3:**
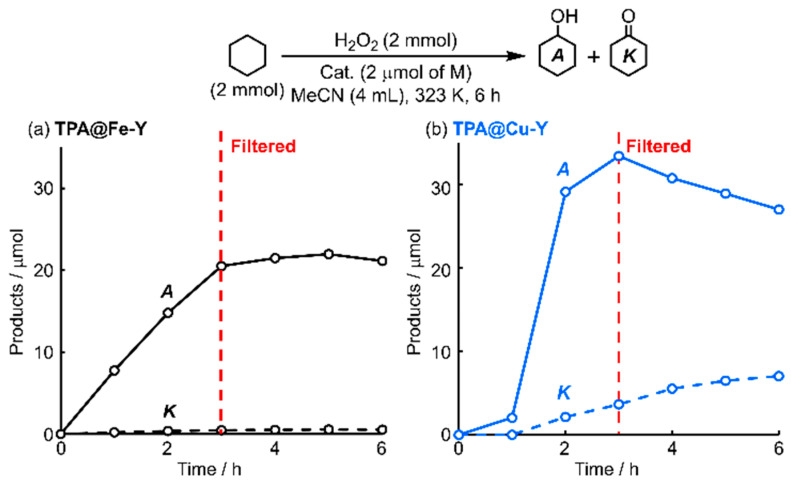
Filtration test on the cyclohexane oxidation catalyzed by TPA@Fe-Y (**a**) and TPA@Cu-Y (**b**).

On the copper catalyst, however, cyclohexanol decreased gradually whereas the ketone increased after 6 h, indicating that the sequential oxidation of alcohol to ketone progressed (Figure S3). In contrast to TPA@Fe-Y, 18% of the copper ions loaded on the initial catalyst leached out into the reaction solvent after 24 h (determined by atomic absorption analysis). To verify the stability of the encapsulated M-TPA species, the catalyst was filtered off from the reaction media 3 h after the start of the reaction and the changing of the yield of products in the filtrate was observed (Figure 3). In the case of TPA@Cu-Y, the alcohol decreased while the ketone increased after catalyst removal. This progressive sequential oxidation indicated that the encapsulated complex was less stable in TPA@Cu-Y and the resulting leached copper species contributed to the oxidation reactions. On the other hand, in the TPA@Fe-Y system, substrate oxidation was almost terminated by the catalyst removal. No iron species were detected by the analysis of the remaining liquid phase. These results of the filtration test supported that TPA@Fe-Y worked as a solid catalyst. Despite the retention of the iron species into the micropore, the UV-vis spectral pattern of the recovered catalyst was not consistent with that of the as-prepared catalyst and the catalytic activity decreased to about half of the initial usage (Figure S4). Therefore, the molecular structure of the encapsulated Fe-TPA complex might be gradually changed during the repetitious action with H_2_O_2_. In contrast, leaching of the immobilized cationic Fe-NHC complex with the chlorine-substituted ligand was observed on the catalyst with the fluoroalkyl-modified mesoporous aluminosilicate support. In this case, the leached complex retained the molecular structure [30]. Notably, the immobilized iron complex with the ligand analogous to TPA, which is covalently anchored into ordered mesopores of SBA-15 type silicate support and the silica wall is modified with the long-chain alkyl fluoride and trimethylsilyl groups, retains the structure and catalytic activity during repetitious usage [29]. These differences in the stability of the immobilized complexes seem to correlate with the immobilization methods, structures of the supports, and the hydrophobic environment of the surrounding space of the complex.

### 2.2. Preparation and Characterization of Mesoporous Zeolite Catalysts

It is known that partial disruption of zeolite particles to impart mesopores increases the number of interfacial micropores that can serve as catalytic active sites [20,21,22,23,24,25,26,27,28,32]. In this study, the performance of the catalysts derived from the prepared mesoporous zeolites was tested to estimate the effect of the mesopore impregnation.

**Figure 4:**
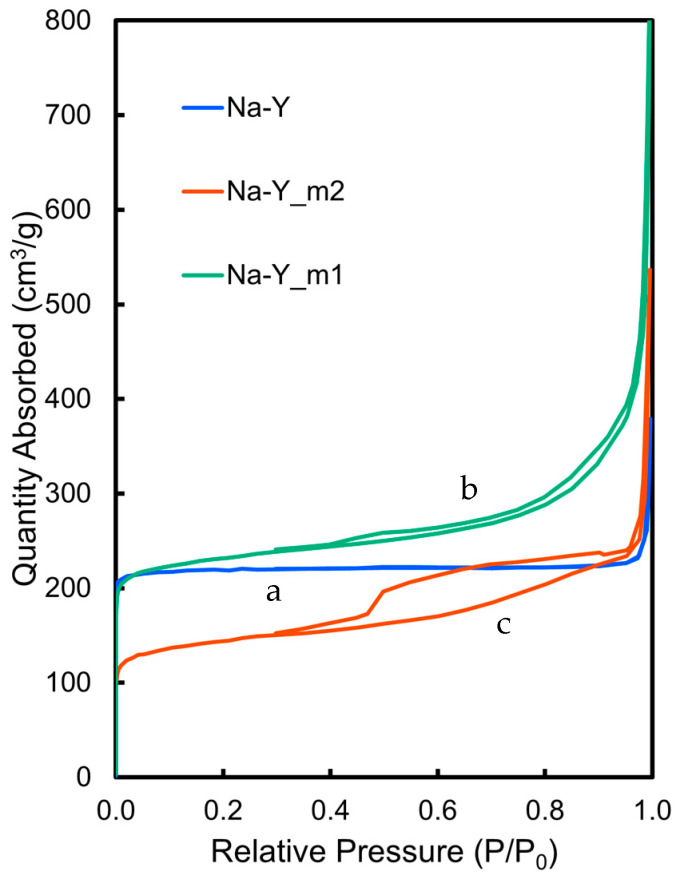
N_2_ adsorption isotherm curves of (a) Na-Y, (b) Na-Y_m1, and (c) Na-Y_m2.

The removal of the part of aluminum ions in the aluminosilicate framework can be achieved by the action of chelating agents, and further alkaline treatment leads to dissolving the silicate producing mesopores in the zeolite particles. In this study, mesoporous zeolites Na-Y_mx were prepared by sequential action of H_4_EDTA, NaOH, and H_2_Na_2_EDTA on Na-Y according to the previous report. One of two mesoporous zeolites, Na-Y_m1, was prepared by dispersing EDTA-treated Na-Y into an aqueous NaOH solution and stirring at 65 °C for 6 h, followed by the hot filtration and treatment of the solid with H_2_Na_2_EDTA [32,33]. Another one, Na-Y_m2, was prepared by standing the suspensions overnight at room temperature without hot filtration. The pore structure of Na-Y and Na-Y_mx was analyzed from N_2_ adsorption/desorption isotherms (Figure 4 and Table 2). The partial collapse of the micropore framework of the zeolite was accompanied by a decrease in surface area and an increase in average pore diameter, suggesting the formation of mesopores. Na-Y_m2 exhibited a clear type IV shape of the N_2_-adsorption/desorption isotherm curve, suggesting that the degree of the mesopore formation was m2 > m1 [34]. Furthermore, Na-Y_m2 adsorbed less nitrogen at a lower relative pressure region, suggesting that the micropores were reduced. In addition, the X-ray powder diffraction pattern of Na-Y_m2 showed a lower peak intensity due to decreasing crystallinity than that of Na-Y_m1, which is consistent with a higher content of mesopores formed through the destruction of micropores (Figure S5). The resulting mesoporous zeolites could be applied to the supports of the ship-in-a-bottle-type catalysts TPA@Fe-Y_mx, which were prepared by the same procedures for the unmodified zeolite catalyst TPA@Fe-Y. As shown in Table 1 (see above), the loading amounts of both iron and TPA in TPA@Fe-Y_mx increased compared to the unmodified zeolite catalyst due to increasing the surface exposed micropores.

The initial activities of the mesoporous zeolite-based catalysts TPA@Fe-Y_m1 and TPA@Fe-Y_m2 were a little different from that of the authentic TPA@Fe-Y, and the catalysts with mesopores showed slightly lower activity at 6 h of reaction. The final order of the activity of the catalysts at 24 h was TPA@Fe-Y_m1 (TON = 44) > TPA@Fe-Y (34) > TPA@Fe-Y_m2 (31) as shown in Figure S6. The Fe-TPA complexes formed in the deeper mesopores were less accessible to substrates and oxidizing agents, resulting in decreased apparent activity. In the case of TPA@Fe-Y, without mesopore formation, the complex molecules working as active sites may not be present in the deep layer of the zeolite. In other words, under the conditions of preparing the catalysts in this study, neither iron ions nor TPA ligands diffused from the surface of the zeolite particles to the deeper regions. This can be interpreted as the absence of the previously reported effect of increased substrate accessibility due to the addition of mesopores [32].

### 2.3. Effect of Surface Modification

We have explored the chemical modification of the zeolite surface with hydrophobic or amphiphilic functional groups because the catalytic activity and reaction selectivity would be varied depending on the affinity of solid catalyst particles and their active sites with oxidants, substrates, and solvents. To investigate the synergistic effect of mesopores and surface modification on catalytic activity, we have also developed new catalysts by the chemical modification of the mesoporous zeolite with organic functionalities.

The surface of the iron catalysts was modified with hydrophobic groups, such as a fluoroalkyl chain (=FC) and trimethylsilyl group (=TMS), by treating the iron catalysts with the corresponding silane-coupling reagent (EtO)_3_SiC_2_H_4_C_6_F_13_ and silanol-capping reagent [Si(CH_3_)_3_]_2_NH, respectively. Considering the presence of water in the liquid phase of the reaction system, (EtO)Me_2_SiC_3_H_6_OCH_2_(CH(-O-)CH_2_) (=E), which is expected to be hydrophilic because it contains two oxygen atoms and the epoxide moiety is easily hydrated giving diol, was also applied as surface modifier. The hydrophilic or hydrophobic nature of the resulting surface modified catalysts TPA@Fe-Y^FG^ and TPA@Fe-Y_mx^FG^ was estimated by the water contact angle (Table S1). On the catalysts modified with E, no water droplets formed, like the unmodified ones (i.e., contact angle was 0°). On the other hand, water droplets formed on the FC or TMS-modified catalysts, and the contact angles were 103–154 deg.

Unfortunately, the surface modification of TPA@Fe-Y was not efficient to improve the catalytic performance. Under the standard reaction conditions (4 mL of MeCN was used as solvent), the extent of the activity loss depended on the molecular size of the grafted functional groups because the order of the activity of TPA@Fe-Y^FG^ was TMS > FC > E on FG. Therefore, the surface modification with the longer chain groups (FC and E) seems to lead to the lower accessibility of the substrate and oxidant to the active site. When the content of water in the solvent was increased (i.e., mixture of 3.5 mL of MeCN and 0.5 mL of H_2_O was used as the solvent), the activity of the E-functionalized catalyst was increased whereas that of the FC-functionalized one was decreased. The activity of the non-functionalized and TMS-functionalized catalysts was also improved by using the water-mixed solvent (Table 3). As reported previously, in the oxidation of benzene with hydrogen peroxide as oxidant over a ship-in-a-bottle-type catalyst with [Fe(bpy)_3_]^2+^ encapsulated in the Y-type zeolite, the activity changed with the volume ratio of both in a mixture of water and acetonitrile, indicating that the application of a mixed solvent system is effective for the proper miscibility of each of the hydrophilic H_2_O_2_ and hydrophobic hydrocarbon substrates [35,36,37]. Therefore, the improvement of activity with the increase in the proportion of water in the mixed solvent for unmodified and TMS-modified catalysts can be attributed to the increased miscibility of H_2_O_2_. For the E- or FC-modified catalysts, the bulky organic groups located near the entrance of the space where the complex molecules are encapsulated. In such catalysts, the hydrophilic or hydrophobic nature of the functional groups were critical to changing the accessibility of H_2_O_2_ to the Fe-TPA species. The water repellent nature of FC prevented the access of aqueous H_2_O_2_ to the catalytic active site, whereas the aqueous H_2_O_2_ might be concentrated near the hydrophilic E groups.

**Figure 5:**
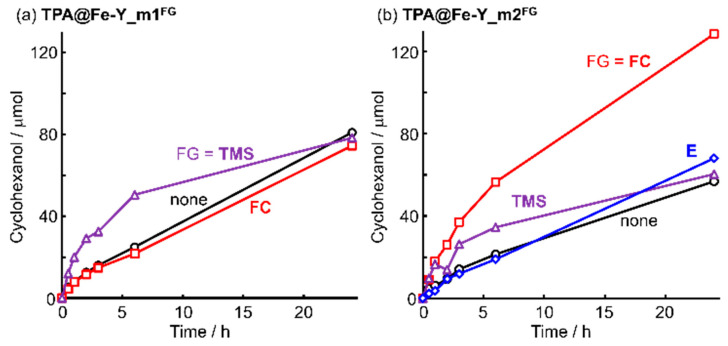
Cyclohexanol production catalyzed by TPA@Fe-Y_mx^FG^ (x = 1 (**a**) and 2 (**b**)).

When the mesopore-impregnated catalyst was applied to the further surface modification with hydrophobic TMS or FC groups, changes in catalytic activity under the standard reaction conditions were observed depending on the mesopore status and the nature of the functional groups. In the case of the TPA@Fe-Y_m1 catalysts (Figure 5a), the TMS-modified one showed a higher initial activity than the non-modified TPA@Fe-Y_m1 and the authentic TPA@Fe-Y. This may be due to the concentration effect of the hydrophobic substrate caused by the modification of the Y_m1 having relatively narrow mesopores with a small TMS. On the other hand, the modification with the long fluoroalkyl chain (FC) showed no improvement in activity because the substrate concentration effect associated with the modification by hydrophobic groups was offset by the steric hindrance of FC.

For the higher degree of the mesopores-formed catalyst TPA@Fe-Y_m2 (Figure 5b), a marked improvement of the catalytic activity was observed in the modification with FC. The mesopores of Y_m2 were enlarged compared to those of Y_m1 and the steric hindrance of FC was relatively reduced. As a result, both the substrate concentration and removal of hydrophilic oxidation products were boosted by the surface modification and these effects might influence the Fe-TPA complex in the micropores facing the mesopores and deep in the mesopores. The TMS-modified catalyst also showed enhanced activity, but not as much as the FC-modified one. No clear increase in initial activity was observed for the hydrophilic groups-functionalized catalyst TPA@Fe-Y_m2^E^. These results indicate that the surface modification of mesoporous supports with hydrophobic functional groups enhances the activity by substrate concentration and accelerating the efflux of oxidation products, similar to the phenomenon observed in the immobilized iron complex catalysts on the mesoporous silica supports developed in our laboratory [29,30]. In the ship-in-a-bottle-type catalysts, this effect was dependent on the structure of the mesopores. The substrate concentration effect exceeded the steric hindrance near the super-cage entrance when the pore size was large. The mesoporosity of the support was derived by the partial destruction of the zeolite backbone structure, and a super-cage with a larger entrance diameter might also be formed facing the mesopore. This situation is similar to the active sites of the immobilized iron complex catalyst on the SBA-15 type mesoporous aluminosilicate support modified with long-chain fluoroalkyl groups [30].

## 3. Materials and Methods

### 3.1. General

Atomic absorption analysis was performed on an AA-6200 (Shimadzu, Kyoto, Japan). UV-vis spectra were measured on a V650 spectrometer with a PIN-757 integrating sphere attachment (JASCO, Tokyo, Japan). Nitrogen sorption/desorption studies were performed at liquid nitrogen temperature (77K) using BELSORP MINI X (MICROTRACBEL, Osaka, Japan). Before the adsorption experiments, the samples were outgassed under reduced pressure for 3 h at 333 K. Thermogravimetric analysis was performed on Thermo plus EVO (Rigaku, Tokyo, Japan). The hydrophilic/hydrophobic properties of the surface were characterized by water contact angle measurements using the Simage AUTO 100 system (Eximer, Yokohama, Japan). Gas chromatography (GC) analyses were conducted on GC-2014 with a flame ionization detector (Shimadzu, Kyoto, Japan) equipped with an Rtx-1701 capillary column (length = 30 m, i.d. = 0.25 mm, thickness = 0.25 μm; Restek, Bellefonte, PA, USA).

The commercially available chemicals were used without further purification.

### 3.2. Preparation of the Catalysts

#### 3.2.1. TPA@M-Y

The metal ion-exchanged Y-type zeolites, M-Y where M = Fe and Cu, were prepared according to the previous reports [2,35]. Prior to the reaction with TPA, M-Y was dispersed in MeOH and the suspension was applied to ultrasound sonication at 333 K for 2 h. To the resulting suspension, 0.33 mmol of TPA per 0.2 g of M-Y was added. Continuous ultrasound sonication at 333 K for 2 h was applied to the resulting reaction mixture. The solid was collected by filtration and washed with MeCN, EtOH, and MeOH. The obtained TPA@M-Y was dried under reduced pressure for 24 h.

#### 3.2.2. TPA@Fe-Y_mx

The mesoporous zeolite Na-Y_m1 was prepared according to the previously reported procedure. Another one, Na-Y_m2 was prepared as follows: To the round-bottomed reaction vessel, 13.4 g of Na-Y, 6.42 g (22.0 mmol) of H_4_EDTA, and 200 mL of deionized water were placed, and the resulting mixture was stirred at 373 K for 6h. After stopping the stirring, the suspension stood at an ambient temperature for 12 h. The white solid was collected and dried by evacuation. The dried solid was dispersed into 0.10 M of aqueous NaOH (200 mL) and stirred at 335 K for 6 h. Then the suspension stood at an ambient temperature for 12 h and the solid was filtered and dried by evacuation. The resulting solid and Na_2_H_2_EDTA (8.21 g, 22.1 mmol) were dispersed in 100 mL of deionized water. The resulting mixture was stirred with heating at 373 K for 6 h and then stood without heating for 12 h. Finally, 8.04 g of Na-Y_m2 was obtained by filtration, washed with H_2_O and dried by evacuation. The pale orange-colored iron-exchanged mesoporous zeolites Fe-Y_mx were prepared by the reaction of Na-Y_mx with an aqueous solution of FeSO_4_∙7H_2_O at ambient temperature for 24 h. The resulting Fe-Y_mx was collected by centrifugation and filtration. To estimate the loadings of Fe, the obtained Fe-Y_mx was dissolved in 3 mL of alkaline H_2_O solution containing a small amount of KOH with heating. The resulting solution was acidified by the addition of HNO_3_ and then diluted with H_2_O. The filtrate was obtained through a membrane filter to analyze by atomic absorption. The reaction of TPA with Fe-Y_mx by the same procedures for the preparation of TPA@Fe-Y yielded the TPA@Fe-Y_mx.

#### 3.2.3. The Surface-Modified Catalysts

The non-functionalized catalyst TPA-Fe-Y or TPA@Fe-Y_mx was dispersed in toluene and stirred at 328 K for 30 min. To this suspension, the silane-coupling reagent for the surface modification was added and then temperature was raised to 343 K. After continuous stirring for 1 h, the modified catalyst was collected by filtration and then washed with appropriate solvents (toluene, CH_2_Cl_2,_ and MeOH for FC and TMS, while EtOH and H_2_O for E). Loading amounts of the functional groups were estimated by the analysis of TG curves.

### 3.3. Catalytic Reaction of Cyclohexane Oxidation

Standard conditions are as follows: In the reaction vessel, the catalyst (as 2 mmol of Fe) was dispersed in 4 mL of MeCN under the air. Then 0.21 mL (2.0 mmol) of cyclohexane (substrate) and 10 mL (0.1 mmol) of nitrobenzene (internal standard for GC analysis) was charged and then warmed at 323 K. Finally, 30 wt% aqueous H_2_O_2_ (0.20 mL, 2.0 mmol) was added to the suspension and the mixture was stirred with 1300 rpm at 323 K for a certain period of time. The products were analyzed by GC measurement after quenching the excess amount of H_2_O_2_ by the addition of PPh_3_.

## 4. Conclusions

The iron complex with tris(pyridylmethyl)amine (=TPA) encapsulated into the micropore of the genuine Y-type zeolite exhibited the selective cyclohexane hydroxylating activity. This ship-in-a-bottle catalyst, TPA@Fe-Y, worked as the heterogeneous catalyst without leaching any iron species, although gradual deactivation occurred due to the structural changing of the iron complex.

The mesoporous zeolites, Na-Y_mx, were prepared by sequential treatment with H_4_EDTA, NaOH, and H_2_Na_2_EDTA on Na-Y. Prolonged exposure to the aqueous NaOH solution after the partial removal of aluminum from the aluminosilicate framework resulted in the higher content of mesopores formed through the destruction of micropores. The catalytic activities of the mesoporous zeolite-based catalysts, TPA@Fe-Y_mx, were slightly lower than that of the genuine zeolite-based catalyst because the Fe-TPA complexes formed in the deeper mesopores were less accessible to substrates and oxidizing agents.

The chemical modification of the zeolite supports with the organic groups led to changing the catalytic activity depending on the size and the hydrophobic or hydrophilic nature of the added organic groups. When the content of water in the solvent was increased, the activity of the hydrophilic longer chain-modified catalyst was improved compared to the one applied on the reaction with the non-aqueous solvent. The hydrophobic fluoroalkyl modifier located near the entrance of the micropore hindered the access of the substrate and aqueous H_2_O_2_ to the encapsulated iron complex site in the genuine Y-type zeolite. However, the hydrophobic modification was effective to improve the activity on the catalyst with the zeolite support having higher amounts of mesopores, demonstrating a synergistic effect of the mesopores and the surface modification with the hydrophobic fluoroalkyl chains by concentrating the hydrocarbon substrate near the active iron complexes located into the mesopores.

These results emphasize the importance of precisely controlling both the structural and chemical properties of the support of the heterogenized metal complex catalyst. The optimal catalyst design requires a balance of factors, including pore structure, surface modification, and the steric environment around the metal complex, to achieve maximum catalytic performance.

## Figures and Tables

**Table 1:** Loading of metal ions (M) and TPA.

Catalyst	Loading Amounts/mmol g^–1 1^		Ratio of M per TPA
	M	TPA	
TPA@Fe-Y	0.112	0.158	0.71
TPA@Cu-Y	0.209	0.212	0.99
TPA@Fe-Y_m1	0.155	0.173	0.90
TPA@Fe-Y_m2	0.131	0.158	0.83

^1^ Quantified by atomic absorption for M and TG for TPA.

**Table 2:** Pysicochemical properties of the zeolites.

Zeolite	Surface Area/m^2^ g^−1^	Average Pore Diameter/nm	Total Pore Volume/cm^3^ g^−1^
Na-Y	909	0.407	1.79
Na-Y_m1	795	0.901	4.53
Na-Y_m2	555	0.555	3.99

**Table 3:** Activity of TPA@Fe-Y^FG^.

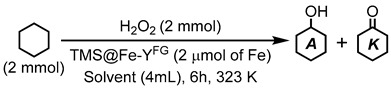					
FG	Solvent ^1^	Products/μmol		*A*/*K*	TON
		*A*	*K*		
None	MeCN	34.5	0.7	53	18
	Mix	97.7	2.2	44	51
FC	MeCN	22.7	0.7	33	12
	Mix	17.2	0.2	86	9
TMS	MeCN	27.4	0.9	31	15
	Mix	75.1	1.5	50	39
E	MeCN	16.8	0.2	84	9
	Mix	75.2	1.6	47	39

^1^ MeCN = 4 mL of MeCN and Mix = 3.5 mL of MeCN + 0.5 mL of H_2_O.

## Data Availability

The data presented in this study are contained within this article and are supported by the data in the Supplementary Materials.

## Supplementary Materials

The following supporting information can be downloaded at: https://www.mdpi.com/article/10.3390/molecules30040966/s1, Figure S1: UV-vis spectra of TPA@M-Y and its precursors; Figure S2: Oxidation of cyclohexane with H_2_O_2_ catalyzed by TPA@Fe-Y; Figure S3: Oxidation of cyclohexane with H_2_O_2_ catalyzed by TPA@Cu-Y; Figure S4: Recycle test on TPA@Fe-Y; Figure S5: PXRD of Na-Y and the mesoporous zeolites Na-Y_mx; Figure S6: Oxidation of cyclohexane with H_2_O_2_ catalyzed by TPA@Fe-Y and TPA@Fe-Y_mx; Table S1: The water contact angles and loadings of the functional groups of TPA@Fe-Y^FG^ and TPA@Fe-Y_mx^FG^.
