# Supplementary material for: Effects of Additional Mesopores and the Surface Modification of the Y-Type Zeolite on the Alkane Oxidation Activity of Iron Complex-Encapsulated Catalysts
Source: Molecules. 2025 Feb 19;30(4):966. doi: 10.3390/molecules30040966 (PMC11858444; doi:10.3390/molecules30040966)
Supplement: Supplementary file 1 [file molecules-30-00966-s001.zip › molecules-3468013-supplementary.pdf]

Supplementary Materials

Effects of additional mesopore and surface modification of the Y-type zeolite on the alkane oxidation activity of iron-complex encapsulated catalysts

Takamasa Takeda,[a] Masaya Okamura,*[a] Syuhei Yamaguchi,[b] Hidenori Yahiro[b] and Shiro Hikichi*[a]

[a] *Department of Applied Chemistry, Faculty of Chemistry and Biochemistry, Kanagawa University, Yokohama, 221-8686, Japan*

[b] *Department of Materials Science and Biotechnology, Graduate School of Science and Engineering, Ehime University, Matsuyama, 790-8577, Japan*

* Corresponding author: hikichi@kanagawa-u.ac.jp (S.H.), okamura@kanagawa-u.ac.jp (M.O.)

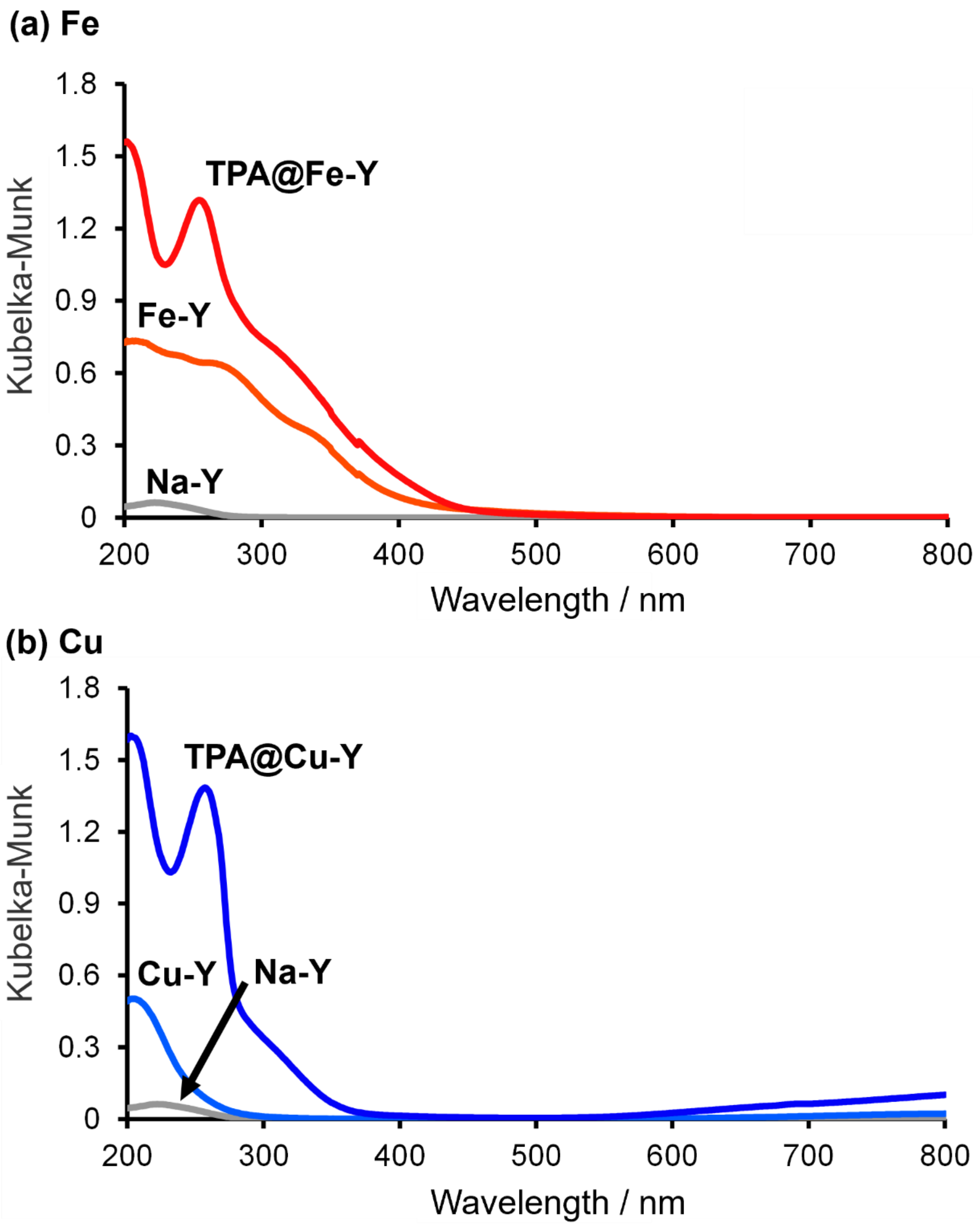

Figure S1. UV-vis spectra of TPA@M-Y and its precursors. Fe (a) and Cu (b).

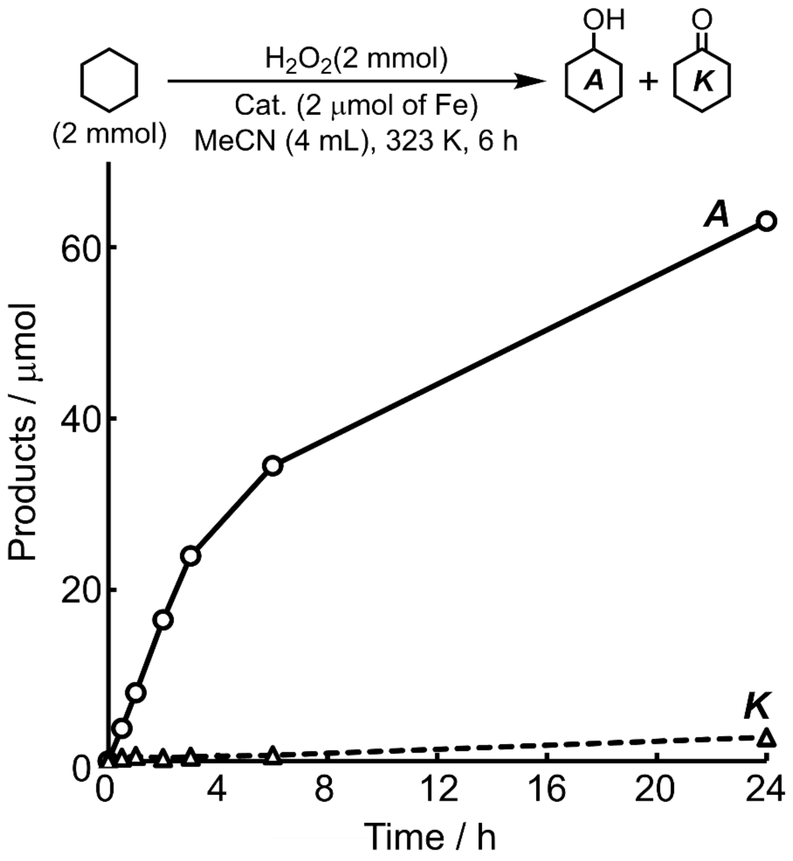

Figure S2. Oxidation of cyclohexane with H_2O_2 catalyzed by TPA@Fe-Y.

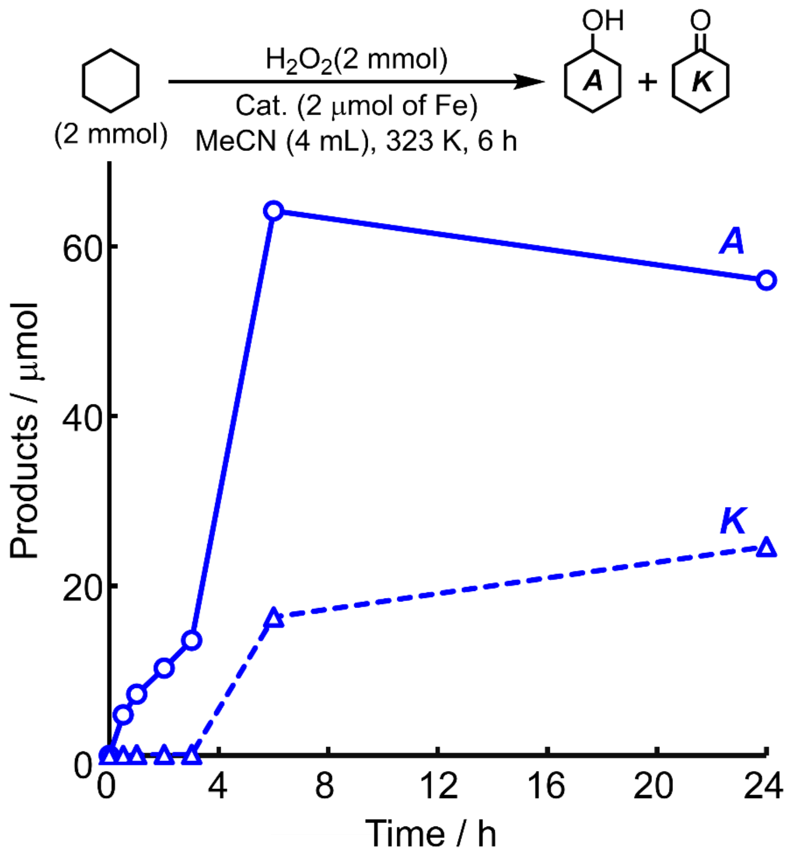

Figure S3. Oxidation of cyclohexane with H_2O_2 catalyzed by TPA@Cu-Y.

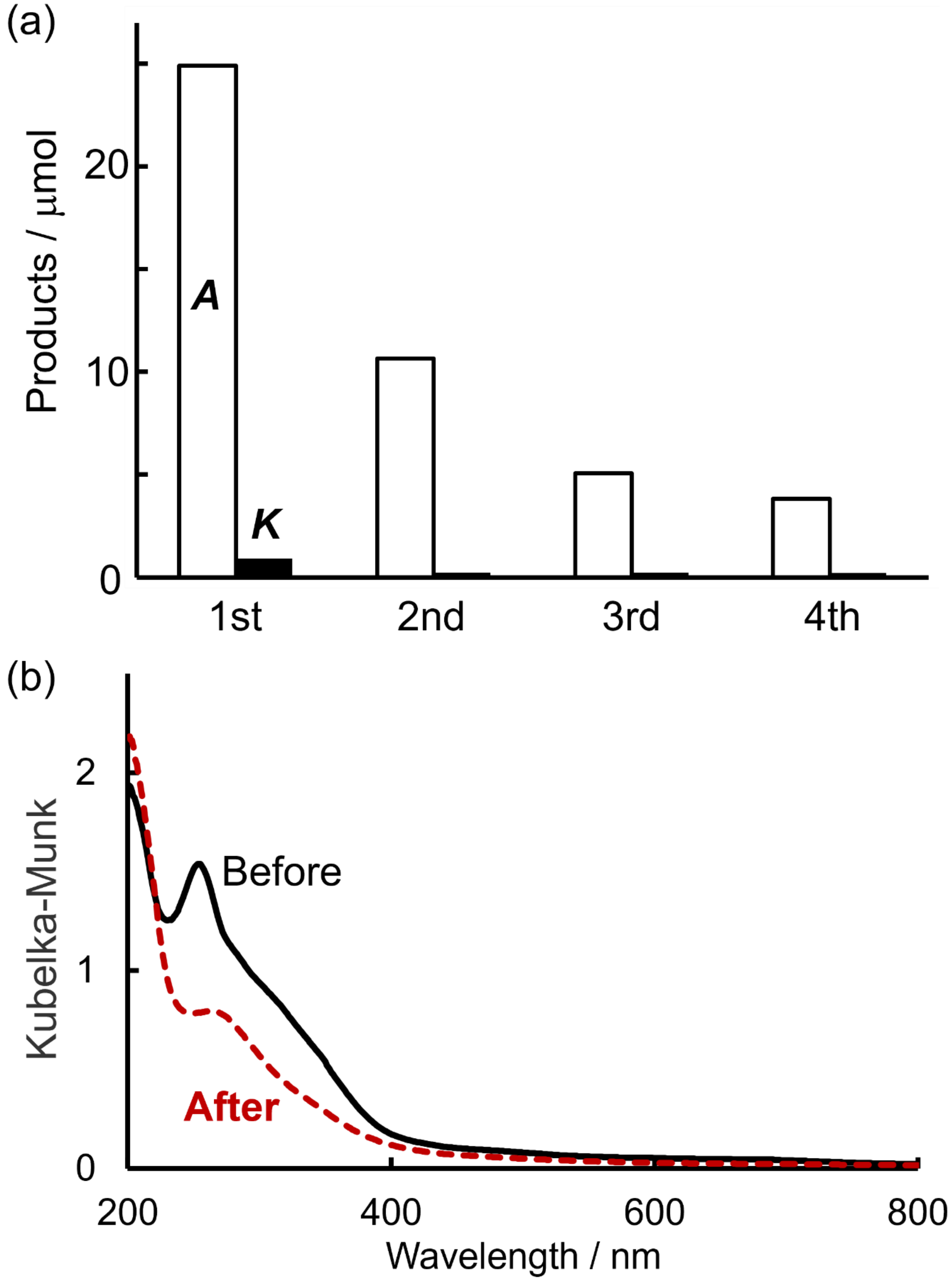

Figure S4. Recycle test on TPA@Fe-Y. Top (a): Changing the products yields on 1st to 4th run. After the 1st run, the used catalyst was recovered by centrifugation and decantation. The recovered catalyst was washed with CH_2Cl_2 and MeCN prior to the next run. Rection conditions: TPA@Fe-Y (2 μmol of Fe at 1st run), cyclohexane (2 mmol), H_2O_2 (2 mmol), MeCN (4 mL), Temperature (323 K), Reaction time (3 h). Bottom (b): UV-vis spectra of before (black line) and after (brown dashed line) usage of 1st run.

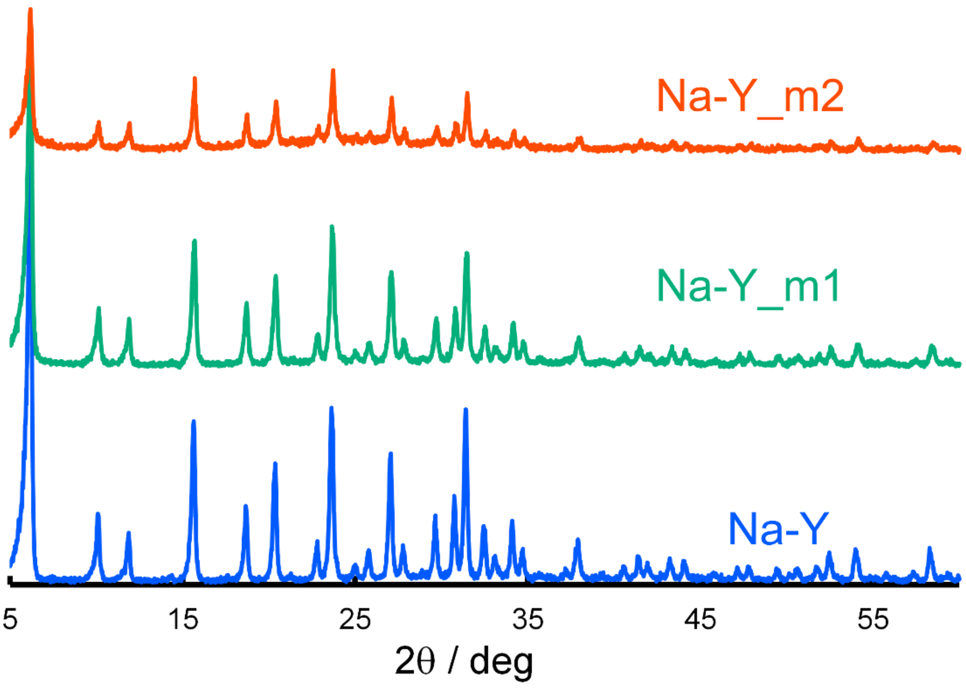

Figure S5. PXRD of Na-Y (bottom) and the mesoporous zeolites Na-Y_mx where x = 1 (middle) and 2 (top).

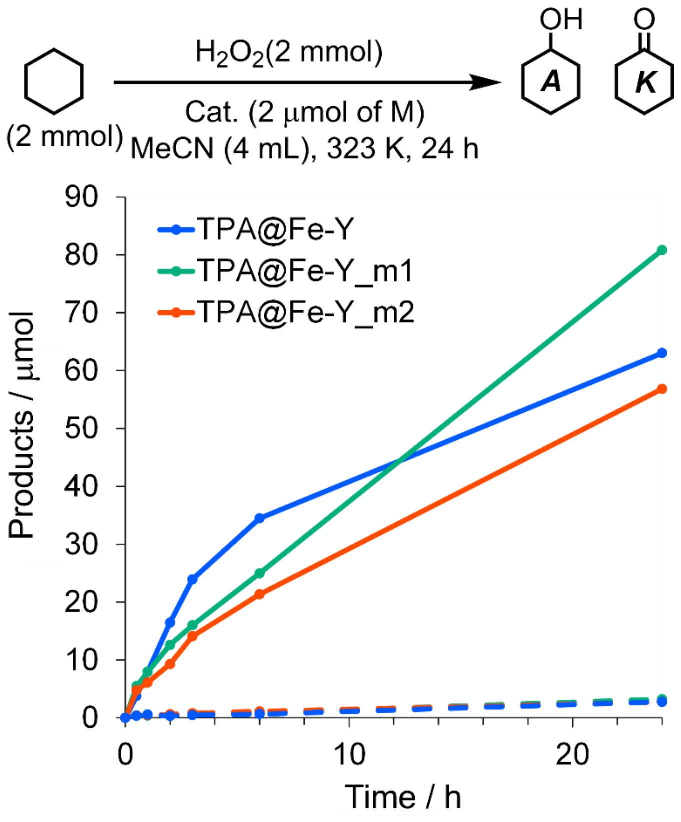

Figure S6. Oxidation of cyclohexane with H_2O_2 catalyzed by TPA@Fe-Y and TPA@Fe-Y_mx (x = 1 and 2).

Table S1. The water contact angles and loadings of the functional groups of TPA@Fe-Y^{FG} and TPA@Fe-Y_mx^{FG}.

Catalyst	FG	Contact angle / deg	Loading of FG / mmol g^{-1}
TPA@Fe-Y^{FG}	FC	154	0.17
	TMS	154	0.75
	E	0	0.37
TPA@Fe-Y_m1^{FG}	FC	103	0.05
	TMS	128	0.71
TPA@Fe-Y_m2^{FG}	FC	128	0.10
	TMS	109	0.49
	E	0	0.69
